# Estimating the Importation Risk of Mpox Virus in 2022 to Hong Kong, China

**DOI:** 10.1155/2023/9943108

**Published:** 2023-08-22

**Authors:** Mingda Xu, Songwei Shan, Zengyang Shao, Yuan Bai, Zhanwei Du, Zhen Wang, Chao Gao

**Affiliations:** ^1^School of Artificial Intelligence, Optics and Electronics (iOPEN), Northwestern Polytechnical University, Xi'an, China; ^2^WHO Collaborating Center for Infectious Disease Epidemiology and Control, School of Public Health, LKS Faculty of Medicine, The University of Hong Kong, Hong Kong SAR, China; ^3^Laboratory of Data Discovery for Health Limited, Hong Kong Science Park, Hong Kong SAR, China; ^4^School of Cybersecurity, Northwestern Polytechnical University, Xi'an, China

## Abstract

International air travel has been recognized as a crucial factor in the cross-regional transmission of monkeypox (now known as mpox) since this disease rapidly spread across the globe in May 2022. On September 6, 2022, Hong Kong SAR (HK) reported its first imported mpox case with travel history of the United States (US), Canada, and the Philippines. In this study, we estimated the importation risk to HK from 25 international departure regions from May 1 to September 6, 2022, based on the prevalence of presymptomatic mpox cases in the study regions, and time-varying flight mobility evaluated by aggregating multiple open-access air travel datasets (e.g., OpenSky and Aviation Edge). The results of the study indicated that during the study period, the highest risk of mpox importation was from the US, at 63% (95% CI: 32% and 95%), followed by the United Kingdom (UK) and Canada, with risks of 29% (95% CI: 10% and 63%), and 17% (95% CI: 8% and 32%), respectively. The importation risk of mpox from the US and Canada was substantially higher than from the other regions, which was aligned with the travel history of the first reported case in HK. Our study introduces a simplified computational method that estimates the risk of importation mpox virus by combining air travel mobility, disease prevalence, and observed real-world scenarios to achieve accurate outcome estimates. Estimating the cross-regional importation risk of mpox would be beneficial in designing and adjusting inbound measures appropriately, which are essential for emergency public health policies.

## 1. Introduction

Mpox (monkeypox) is a zoonotic disease rarely transmitted outside Africa before 2022 [1–3]. On May 7, 2022, a confirmed case of mpox who traveled from Nigeria to the United Kingdom (UK) [4] was reported by the World Health Organization. After that, the rapid outbreak marked the first time that mpox spread widely in non-endemic countries, covering Europe, North America, and Oceania successively [5]. On July 23, 2022, World Health Organization declared the ongoing mpox outbreak as a Public Health Emergency of International Concern (PHEIC) [6]. As of December 28, 2022, 83,751 cases had been reported in 110 countries or regions, with 9 countries reporting a high-cumulative number of cases (≥3,000), namely the US (*n* = 29,554), Brazil (*n* = 10,493), Spain (*n* = 7,496), France (*n* = 4,114), Colombia (*n* = 4,021), UK (*n* = 3,730), Germany (*n* = 3,676), Peru (*n* = 3,643), and Mexico (*n* = 3,509) [7].

International air travel plays a critical role in the transmission of mpox [8]. Most confirmed cases were with travel history from European and North American countries rather than West or Central Africa [9, 10]. The first imported mpox case in Hong Kong SAR (HK), whose recent stops included the US, Canada, and the Philippines, was documented on September 6, 2022 [11]. The Government of Hong Kong has procured a third-generation vaccine, “JYNNEOS,” which has been licensed by the US Food and Drug Administration (FDA) to protect against mpox [12]. Although rapid responses were quickly initiated in non-endemic areas, including vaccination and surveillance of human-to-human transmission, travelers from high-risk regions may still pose a potential importation risk to the destinations due to the long incubation period [13].

Various mathematical models and research methods have been developed to estimate the risk of cross-regional transmission. Previous research has provided vital insight into infectious disease transmission by combining travel data with local disease surveillance data [14–17]. In the fight against COVID-19 outbreaks, the importation risk of COVID-19 (including the wildtype, 501Y, and Omicron subvariants) is quantified based on the accessibility to the airline network and local disease situations to guide inbound control measures [18–21]. The correlations between air travel mobility and the international transmission of mpox have been clearly identified [22–24]. These studies highlighted the importance of appropriately estimating the importation risk among air passengers to effectively mitigate the cross-regional transmission of infectious disease [25–27]. However, previous studies have shown that there were biases in the estimation of disease importation risk due to limited data sources [28, 29]. Making more reasonable assumptions and integrating multiple data sources can further improve the accuracy of emergency importation risk estimations for the specific regions.

As a major international transportation hub and a special administrative region of China, HK has been affected by the global spread of infectious diseases over the past decades [3, 30]. HK experienced the SARS outbreak in 2003 [31] as well as the Influenza A (H1N1) pandemic in 2009 [32], and further fueled and accelerated the global transmission of these diseases [33]. The air traffic from overseas to Hong Kong has been increasing gradually following the reopening of HK on August 12, 2022 [34]. Our study aims to quantify the importation risk of mpox by combining aviation transportation data and prevalence in origin regions estimated during the early stage of the emergency public health outbreak, using HK as a case study. We estimated the daily volumes of air travel passengers from international regions to HK via open-access aviation datasets (e.g., OpenSky [35], Aviation Edge [36], and the prevalence of mpox by country from Our World in Data [7]. Our research included 25 regions covering four continents with the most reported cases and direct air connection to HK. We further estimated the likelihood of importing at least one case of mpox, taking into account the number of observed cases before September 6, 2022, which was the date of the first imported case of mpox in HK. Understanding and nowcasting the international importation risk of the mpox virus would be critical for designing appropriate emergency public health strategies and inbound measures to reduce the transmission risk.

## 2. Materials and Methods

### 2.1. Data Source

To estimate the daily number of passengers traveling to HK, we aggregated the daily direct flights arriving (and landing) in HK by combining the results from the OpenSky [35] and Aviation Edge [36]. The information for each airport and estimated passenger capacity per aircraft were obtained from OurAirports [37] and the Admtl [38], respectively.

We retrieved monthly data for about 696,000 air-travel passengers arriving or transiting in HK during the study period *T* (from May 1 to September 6, 2022) [39]. In total, we extracted 20,181 direct and transit flights from 46 distinct regions, with 14,858 from OpenSky and 5,323 additional from Aviation Edge. Due to the lack of transit flight information, we assumed HK was the destination for every flight landing at Hong Kong International Airport. We obtained the 7-day smoothed daily reported cases of mpox of different countries and regions from Our World in Data [7] from May to September 2022.

### 2.2. Estimating Daily Air-Travel Passengers from International Regions to HK

We first estimate the time-varying mobility via air travel following the methods used by Bai et al. [40] and then calculate the prevalence of presymptomatic cases in a given area to estimate the likelihood of introductions. The notation and values of the parameters are provided in Table 1.

Given the lack of information regarding the actual composition of each flight, it was assumed that each flight was at full capacity. Used *Ω*_*o*_^*a*^(*t*) is the daily volume of aircraft *a* from the international region *o* to HK and *ϵ*_*a*_ is the maximum passenger capacity for aircraft *a* of all types of aircraft *A* [38]. Thus, the approximated inbound passengers *m*_*o*_(*t*) from *o* to HK on day *t* is given by: $m_{o}\left(t\right)=\sum_{a,a\in A}\Omega_{o}^{a}\left(t\right)\times \epsilon_{a}$. Then, we validated the air traffic by defining the ascertainment rate of passenger volume *Υ*_*o*_(*T*) as the ratio of actual and estimated passengers. The ascertainment rate will help us to adjust for any potential overestimation of the volume of air travelers, which is given by the following:

(1) $$\begin{array}{c} \Upsilon_{o}\left(T\right)=\frac{\delta_{o}\left(T\right)}{\sum_{t,t\in T}m_{o}\left(t\right)}, \end{array}$$

where *δ*_*o*_(*T*) stands for the actual passengers from *o* during *T*. For those regions without the monthly visitor statistics to HK [39], we matched *m*_*o*_(*t*) and the ascertainment rate *Υ*'_*o*_(*T*) to estimate the daily inbound volume: *M*_*o*_(*T*) = *Υ*'_*o*_(*T*) × *m*_*o*_(*t*).

### 2.3. Estimating Presymptomatic Prevalence of Mpox

We utilized 7-day smoothed mpox cases reported by region [7] to estimate the presymptomatic prevalence of mpox. Following the subexponential model [23, 43], we first estimated the confirmed case on day *t* during the early outbreak of epidemics by:

(2) $$\begin{array}{c} I_{o}\left(t\right)=r{\left[r\left(1-p\right)t+I_{o}{\left(t_{o}\right)}^{\left(1-p\right)}\right]}^{\left(1/1-p\right)}, \end{array}$$

where *t*_*o*_ indicates the date when the case was first detected in region *o* and *I*_*o*_(*t*_*o*_) the corresponding initial number, *r* and *p* are the growth rate and the deceleration of growth, respectively. We assume *r* and *p* follow the normal distributions and estimate their mean and standard variance based on the daily confirmed cases in the study region, using the Levenberg–Marquardt algorithm implemented in the prior studies [43] to minimize the residual sum of squares (RSS) between the model output and the observed cases.

To calculate the total number of infected cases who had not yet developed symptoms *Φ*_*o*_(*t*), we back-shifted the time series of reported cases by incubation period *D*_*i*_:

(3) $$\begin{array}{c} \Phi_{o}\left(t\right)=\sum_{i=t-D_{i}}^{t-1}I_{o}\left(i\right). \end{array}$$

Then, we divided the total cases by the population size *N*_*o*_ of the origin *o* to calculate the prevalence of presymptomatic cases, which is given by *P*_*o*_(*t*) = *Φ*_*o*_(*t*)/*N*_*o*_. For each simulation to yield the incidence in the region *o* during the study period, we independently simulate 1,000 times to sample a pair of *r* and *p* from the normal distributions to introduce uncertainty.

### 2.4. Estimating Importation Risk of Mpox

We assumed all the mpox cases (had not developed symptoms among passengers who intended to travel to HK) would be able to board. To estimate the number of imported cases from each region, we combined air passenger with the daily prevalence of mpox to yield the importation force of cases that have not developed symptoms. We assumed that the proportion of presymptomatic passengers departing from a region was the same as the overall prevalence of presymptomatic cases in that region. Thus, the imported cases from region *o* to HK on day *t*, is given by:

(4) $$\begin{array}{c} \Gamma_{o}\left(t\right)=P_{o}\left(t\right)\times M_{o}\left(t\right). \end{array}$$

Informed by the first case among inbound passengers reported in HK who might be infected in the US on September 6, 2022 [11], we calibrated the cumulative number of imported cases from the US as one at the end of the study period. Based on the observed event by September 6, 2022, we normalized each origin region's cumulative imported cases, by ${\hat{\Gamma }}_{o}\left(t\right)=\Gamma_{o}\left(t\right)/\Gamma_{US}\left(t_{n}\right)$, where Γ_*US*_(*t*_*n*_) = 1 denotes the observed imported cases from the US on *t*_*n*_ (September 6, 2022).

Assuming that the imported cases of mpox from the study region *o* to HK are essentially a nonhomogeneous Poisson process [44, 45], we estimated the cumulative probability of at least one infected case [20, 28] being introduced from *o* to HK by *t* as:

(5) $$\begin{array}{c} \Psi_{o}\left(t\right)=1-\exp\left[-\int_{i=t_{0}}^{t}{\hat{\Gamma }}_{o}\left(i\right)di\right]. \end{array}$$

## 3. Results

We estimated the time-varying flight mobility from a given country or region to HK with multiple open-access air travel datasets and the prevalence of presymptomatic cases in each study region. We synthesized these results to calculate the likelihood of at least one imported case occurring before September 6, 2022. Two indicators for the international importation risk of mpox to HK, namely the cumulative volume of imported cases and the cumulative probability of importing at least one case, were estimated by combining the aviation transportation data and the prevalence in origin regions. The estimated importation risk for the 25 regions between May 1 and September 6, 2022 is shown in Table 2.

**Figure 1:**
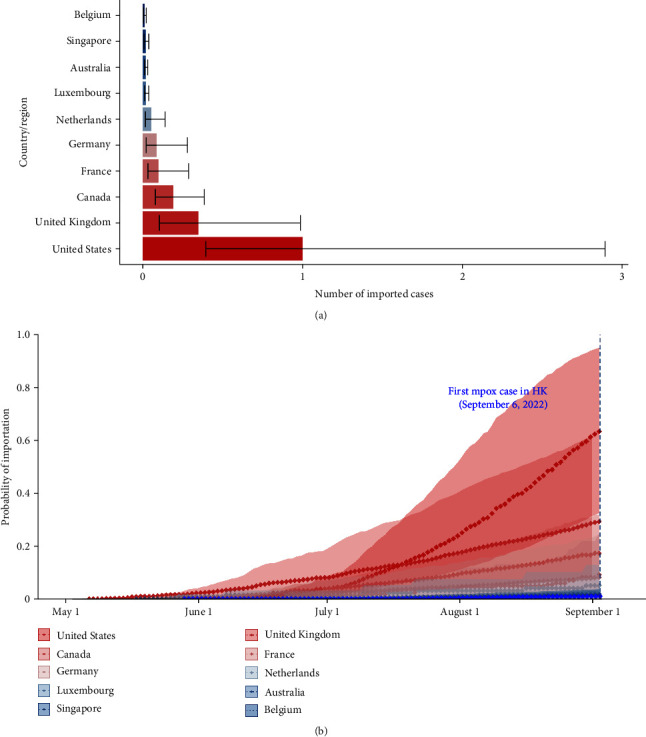
Estimated risk of mpox introductions from international regions to HK via 1,000 stochastic simulations from May 1 to September 6, 2022. (a) As of September 6, 2022, the estimated number of imported cases arrived in Hong Kong based on OpenSky data and Aviation API database. The barplot shows the estimated number of imported cases from the top 10 regions during the study period, along with a 95% confidence interval; (b) probability that > 1 individual infected with mpox virus imported to Hong Kong SAR from the study countries and regions by the date indicated on the *x*-axis. The dotted blue vertical line indicates September 6, 2022, the date when the first mpox case was reported in Hong Kong SAR. The recent stops, including the US, Canada, and the Philippines, are shown in bold. Line colors correspond to the relative risk for importations as of that date, with red and blue denoting high and low probability, respectively.

Figure 1(a) shows the estimated number of imported cases in HK by September 6, 2022. The *x*-axis designates the estimated cumulative imported cases. Among the 25 international regions included in this study, we estimated that the US imported the maximum cases at 1.0 (95% CI: 0.39 and 2.89), followed by UK at 0.35 (95% CI: 0.10 and 0.99) and Canada at 0.19 (95% CI: 0.08 and 0.38).

Figure 1(b) represents the curve of importation risk reflecting the cumulative probability of importing at least one mpox case among inbound travelers. Based on the observed sources of imported cases, only the US had a mean importation risk of 63% (95% CI: 32% and 95%) higher than 50% as of September 6, 2022. The UK and Canada are the following, with 29% (10% and 63%) and 17% (8% and 32%), respectively.

**Figure 2:**
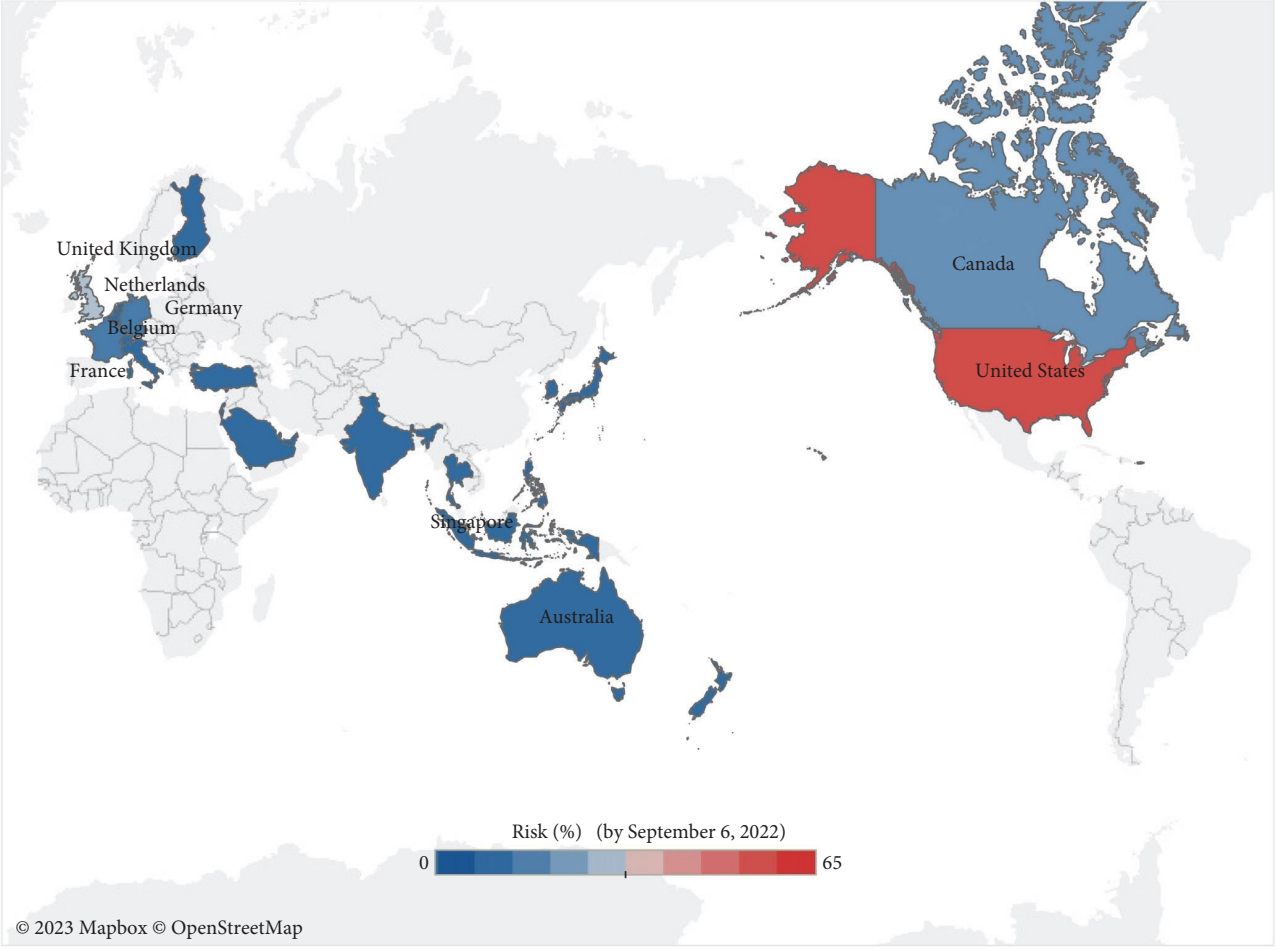
Cumulative probability of at least one mpox case imported from international regions to HK by September 6, 2022 in 1,000 stochastic simulations. Red to blue indicate the regions with high and low risk, respectively. Regions shaded in gray were not analyzed due to the lack of available mobility data or the absence of reported cases of mpox.

Figure 2 displays the risk of importation of mpox for the study regions as of September 6, 2022. Regions with colors closer to red indicate a higher risk of importation, while regions shaded in gray were not analyzed due to a lack of available mobility data or the absence of reported cases of the mpox.

## 4. Discussion

Understanding human mobility patterns would be beneficial for measuring and mitigating epidemiological risks [46]. It is important to be aware of travel-related infections and the potential for global travelers to disseminate the infection and introduce pathogens to previously unaffected areas [47]. The source of importation of a novel emerging infectious disease will most likely be determined by the high prevalence and incidence at potential departure locations for international travel [16]. Drawing upon two strands aforementioned of research into risk estimation, this study attempts to investigate the time-varying importation risk of mpox from international regions to HK. Informed by the arrival number of passengers and the local prevalence of mpox in each international departure location, we developed a statistical method to estimate the cumulative risk of importation of mpox by combining actual imported events in the HK. Our results were aligned with reality, and the estimates of imported cases reflected the connectivity of air travel and the prevalence of mpox in the corresponding origin countries.

Our findings emphasize the importance of enhancing inbound surveillance among travelers from regions with a high prevalence of mpox. The cumulative risk of importation from a region is highly correlated with the aggregated air travel flow and the prevalence of mpox. Our study found that the risk from the US and Canada was relatively higher, which is aligned with the facts of observation that the first reported case in HK once stayed in both countries before arriving and may have been infected there. The counts of mpox cases in the US surged from July to August [48], and the volume of travelers from the US continuously increased during this period due to the lifting of boarding requirements and inbound compulsory in HK [39, 34]. A previous study by Du et al. [23] utilizing the risk matrix method arrived at a similar conclusion regarding the importation risk of mpox into Mainland China: the US posed the highest risk of mpox importation and should be subject to close monitoring. Except for the US, where the imported cases have been observed, several non-endemic countries, including the UK, Canada, France, Germany, and the Netherlands, are also at relatively high risk of importation. During the current outbreak, mpox virus is typically transmitted through MSM (men who have sex with men) [49], close contact with groups who developed symptoms, such as healthcare workers, laboratory personnel, and individuals who engage in “high-risk sexual practices”, such as sex workers [50]. Although the probability of importing a case of mpox via international air travel is extremely low at the end of August 2022, implementing health surveillance and contact tracing for passengers with potential high-risk activities from specific areas would effectively contain the cross-regional transmission of mpox.

Several assumptions and limitations should be discussed in our methods and estimations. First, due to unavailability of the fine-resolution dataset of commercial aviation, we postulated that air travel between regions is only directed, while the indirect travels should also be considered in the air traffic volumes. Owing to the lack of information regarding the proportion of transit passengers from certain countries in HK International Airport in 2022, these initial assumptions could lead to an overestimation of the overall travel volume. To mitigate these biases, adjustments were made to the number of daily visitors from each country using Hong Kong tourism statistics, ensuring the accuracy of the overall volume of air passengers without compromising data quality. However, misidentifying risk sources can result in ignoring the risk of importation in specific areas. Combining complete passengers' travel information with flight transfer records is expected to improve the accuracy of estimates of absolute importation risks from international departures, but would not expect to substantially change the analysis results. Our main contribution to this analysis is the relative risk based on the observed importation events. As a result, the risk indicators presented in this article do not provide absolute estimates of the importation risk.

Second, our approach of estimating the disease prevalence relies on the reported cases in the early phase of an outbreak. However, by giving the differences in mpox surveillance capacity across various regions and the delay between infection and case reporting [51], undetected cases may lead to underestimation of local presymptomatic prevalence. Furthermore, we assumed that only infected cases who had not developed symptoms could board following the study by Du et al. [28]. While in reality, individuals with asymptomatic or mild infection may also be able to go aboard [52]. Understanding the contribution of asymptomatic individuals to the transmission of mpox disease is critical in improving our estimates of potentially infected individuals among air travelers [53, 54].

Last, our approach only applied to the importation risks posed by the air traffic. Hence the results cannot capture the risk of importation between regions with the high volumes of land and marine traffic [16, 25, 55].

## 5. Conclusions

To conclude, our study provided a simplified computational method for estimating the mpox virus importation risk based on air travel mobility and disease prevalence. The approach can be adjusted to incorporate prevalence estimates and flight data from any origin and destination locations requiring minimum data. Our findings emphasize the necessity for a more comprehensive understanding of the probable sources of case importation for predictive simulation and risk perception. Reliable estimates of the risk of international importation from endemic or non-endemic areas will benefit the accurate quantification of the potential need to curtail cross-regional transmission of the pox virus, as well as the implementation of disease monitoring and preparedness.

## Figures and Tables

**Table 1:** Model parameters and data sources.

Symbol	Description	Values	Sources
*Ω*_*o*_^*a*^(*t*)	Number of landed flights of type *a* from region *o* to HK at time *t*	Daily inbound flights	[35]
*ϵ*_*a*_	Passenger capacity of aircraft *a*	Capacity of each aircraft	[38]
*δ*_*o*_(*T*)	Number of HK daily inbound passengers during study period *T*	Monthly inbound passengers	[39]
*Υ*_*o*_(*T*)	Ascertainment rate of air traffic of region *o*	Scaling factor	Estimated
*M*_*o*_(*t*)	Volume of estimated air passengers from region *o* to HK at time *t*	Daily air passengers	Estimated
*N*_*o*_	Population size of region *o*	2021 Population estimates	[41]
*I*_*o*_(*t*)	The number of new reported cases per person in the general population per unit time	Incidence	Estimated
*P*_*o*_(*t*)	The prevalence of presymptomatic mpox cases	Prevalence (rate)	Estimated
*D*_*i*_	The time period between infection and symptoms onset	Incubation period 7 days (range: 3, 20)	[13]
Γ_*o*_(*t*)	Importation force from the region/country *o* at time *t*	Estimated volume of imported cases	Estimated
Ψ_*o*_(*t*)	The cumulative probability of imported at least one infection from the region *o* during study period *T*	Estimated importation risk	Estimated

**Table 2:** Importation risk estimated during the study period.

Location	Importation risk (95% CI)
**United States**	**63.21% (32.41%, 94.73%)**
United Kingdom	29.17% (9.80%, 62.71%)
**Canada**	**17.27% (7.55%, 31.76%)**
France	9.21% (3.15%, 24.75%)
Germany	8.18% (2.09%, 24.21%)
Netherlands	5.10% (1.61%, 12.91%)
Luxembourg	1.82% (0.94%, 3.61%)
Australia	1.73% (1.04%, 3.05%)
Singapore	1.70% (0.77%, 3.67%)
Belgium	1.05% (0.48%, 2.07%)
Switzerland	0.50% (0.28%, 0.86%)
Israel	0.45% (0.25%, 0.76%)
Italy	0.32% (0.14%, 0.73%)
Qatar	0.24% (0.13%, 0.44%)
New Zealand	0.11% (0.06%, 0.21%)
United Arab Emirates	0.10% (0.01%, 0.36%)
Thailand	0.06% (0, 0.10%)
Finland	0.04% (0.01%, 0.12%)
**Philippines**	**0.01% (0, 0.02%)**
Japan	0.01% (0.01%, 0.02%)
South Korea	0.01% (0, 0.02%)
Saudi Arabia	0 (0, 0.01%)
India	0 (0, 0)
Turkey	0 (0, 0)
Indonesia	0 (0, 0)

*Note:* We estimated the cumulative risk of importation (95% CI) from 25 regions as of September 6, 2022. The region in bold denotes the first case detected in HK who arrived from the Philippines after traveling in the US and Canada.

## Data Availability

The data underlying this article and these programs will be shared on reasonable request with the corresponding author (email to the corresponding author). The database of the air travel and mpox cases is publicly available at crowdsourced air traffic data from The OpenSky Network 2020 | Zenodo, Aviation Edge—Database, and API—Aviation database and API (https://aviation-edge.com), Mpox (monkeypox)-Our World in Data, respectively.

## References

[1] (2022). Monkeypox: wealthy countries must avoid their COVID-19 mistakes. *Nature*.

[2] Wang S., Zhang F., Yuan Z. (2022). Serial intervals and incubation periods of the monkeypox virus clades. *Journal of Travel Medicine*.

[3] Du Z., Tian L., Jin D.-Y. (2022). Understanding the impact of rapid antigen tests on SARS-CoV-2 transmission in the fifth wave of COVID-19 in Hong Kong in early 2022. *Emerging Microbes & Infections*.

[4] World Health Organization (2022). Disease outbreak news; monkeypox—United Kingdom of Great Britain and Northern Ireland. https://www.who.int/emergencies/disease-outbreak-news/item/2022-DON381.

[5] World Health Organization (2022). Disease outbreak news; multi-country monkeypox outbreak in non-endemic countries. https://www.who.int/emergencies/disease-outbreak-news/item/2022-DON385.

[6] WHO Director-General Declares the Ongoing Monkeypox Outbreak a Public Health Emergency of International Concern (2022). https://www.who.int/europe/news/item/23-07-2022-who-director-general-declares-the-ongoing-monkeypox-outbreak-a-public-health-event-of-international-concern.

[7] Mathieu E., Spooner F., Dattani S., Ritchie H., Roser M. (2022). Monkeypox.. *Our World in Data*.

[8] Talbot F. (2022). The impact of COVID-19 on airports—and the path to recovery, *ACI World* (blog). https://aci.aero/2022/10/06/the-impact-of-covid-19-on-airports-and-the-path-to-recovery/.

[9] Monkeypox Outbreak (2022). https://www.who.int/emergencies/situations/monkeypox-oubreak-2022.

[10] Angelo K. M., Petersen B. W., Hamer D. H., Schwartz E., Brunette G. (2019). Monkeypox transmission among international travellers—serious monkey business?. *Journal of Travel Medicine*.

[11] Reuters (2022a). Hong Kong discovers first case of monkeypox. https://www.reuters.com/world/asia-pacific/hong-kong-discovers-first-case-monkeypox-2022-09-06/.

[12] Centre for Health Protection, Department of Health—Monkeypox (2022). https://www.chp.gov.hk/en/healthtopics/content/24/101721.html.

[13] Thornhill J. P., Antinori A., Orkin C. M. (2022). Monkeypox virus infection in humans across 16 countries—April-June 2022. Reply. *The New England Journal of Medicine*.

[14] Shi S., Tanaka S., Ueno R. (2020). Travel restrictions and SARS-CoV-2 transmission: an effective distance approach to estimate impact. *Bulletin of the World Health Organization*.

[15] Kuo P.-F., Chiu C.-S., Wang J. (2021). Airline transportation and arrival time of international disease spread: a case study of COVID-19. *PLoS One*.

[16] Russell T. W., Wu J. T., Sam Clifford W. J. E., Kucharski A. J., Jit M., Centre for the Mathematical Modelling of Infectious Diseases COVID-19 working group (2021). Effect of internationally imported cases on internal spread of COVID-19: a mathematical modelling study. *Lancet Public Health*.

[17] Zhang L., Zhang L., Lai L. (2022). Risk assessment of imported COVID-19 in China: a modelling study in Sichuan province. *Transboundary and Emerging Diseases*.

[18] Nakamura H., Managi S. (2020). Airport risk of importation and exportation of the COVID-19 pandemic. *Transport Policy*.

[19] Du Z., Wang L., Cauchemez S. (2020). Risk for transportation of coronavirus disease from Wuhan to other cities in China. *Emerging Infectious Diseases*.

[20] Bai Y., Xu M., Liu C. (2022). Travel—related importation and exportation risks of SARS-CoV-2 Omicron variant in 367 prefectures (cities)—China. *China CDC Weekly*.

[21] Xu M., Du Z., Shan S. (2022). RiskEstim: a software package to quantify COVID-19 importation. *Frontiers in Physics*.

[22] Bhattacharya M., Dhama K., Chakraborty C. (2022). Recently spreading human monkeypox virus infection and its transmission during COVID-19 pandemic period: a travelers’ prospective. *Travel Medicine and Infectious Disease*.

[23] Du M., Zhang S. M., Shang W. J. (2022). 2022 Multiple-country monkeypox outbreak and its importation risk into China: an assessment based on the risk matrix method. *Biomedical and Environmental Sciences*.

[24] Kinoshita R., Sassa M., Otake S. (2023). Impact of airline network on the global importation risk of mpox, 2022. *Epidemiology & Infection*.

[25] Menkir T. F., Chin T., Hay J. A. (2021). Estimating internationally imported cases during the early COVID-19 pandemic. *Nature Communications*.

[26] Lai S., Bogoch I. I., Ruktanonchai N. W. (2022). Assessing spread risk of COVID-19 within and beyond China in early 2020. *Data Science and Management*.

[27] Han X., Xu Y., Fan L., Huang Y., Xu M., Gao S. (2021). Quantifying COVID-19 importation risk in a dynamic network of domestic cities and international countries. *Proceedings of the National Academy of Sciences*.

[28] Du Z., Wang L., Yang B. (2021). Risk for international importations of variant SARS-CoV-2 originating in the United Kingdom. *Emerging Infectious Diseases*.

[29] Du Z., Shao Z., Bai Y. (2022). Reproduction number of monkeypox in the early stage of the 2022 multi-country outbreak. *Journal of Travel Medicine*.

[30] Du Z., Zhang X., Wang L. (2023). Characterizing human collective behaviors during COVID-19—Hong Kong SAR, China, 2020. *China CDC Weekly*.

[31] Pine R., McKercher B. (2004). The impact of SARS on Hong Kong’s tourism industry. *International Journal of Contemporary Hospitality Management*.

[32] Liao Q., Cowling B., Lam W. T., Ng M. W., Fielding R., Ng L. F. P. (2010). Situational awareness and health protective responses to pandemic influenza a (H1N1) in Hong Kong: a cross-sectional study. *PLoS One*.

[33] National Immunization Advisory Committee (NIAC) Technical Working Group (TWG), Influenza Vaccination TWG (2021). Technical guidelines for seasonal influenza vaccination in China (2021–2022). *Zhonghua liu xing bing xue za zhi = Zhonghua liuxingbingxue zazhi*.

[34] Government Announces Lifting of Compulsory Quarantine Requirement on Arrival at Hong Kong (2023). https://www.info.gov.hk/gia/general/202209/24/P2022092400048.htm.

[35] Meides, Marco (2022). The opensky network—free ADS-B and mode S data for research. https://opensky-network.org/.

[36] Aviation API List (2017). Aviation database and API. Aviation edge. https://aviation-edge.com/aviation-api-list/.

[37] Open Data @ OurAirports (2022). https://ourairports.com/data/.

[38] Aéroports de Montréal (2021). Reference table —number of seats per aircraft type. https://www.admtl.com/sites/default/files/2021/2021_Appendix_6-Number-of-seats-per-aircraft-type.pdf.

[39] “Tourism Statistics.” (2022). Discover Hong Kong. https://www.discoverhongkong.com/eng/hktb/newsroom/tourism-statistics.html.

[40] Bai Y., Du Z., Xu M. (2022). International risk of SARS-CoV-2 Omicron variant importations originating in South Africa. *Journal of Travel Medicine*.

[41] World Population Prospects—Population Division—United Nations (2022). https://population.un.org/wpp/Download/Standard/CSV/.

[42] Chowell G., Viboud C., Simonsen L., Moghadas S. M. (2016). Characterizing the reproduction number of epidemics with early subexponential growth dynamics. *Journal of the Royal Society Interface*.

[43] Viboud C., Simonsen L., Chowell G. (2016). A generalized-growth model to characterize the early ascending phase of infectious disease outbreaks. *Epidemics*.

[44] Wang L., Wu J. T. (2018). Characterizing the dynamics underlying global spread of epidemics. *Nature Communications*.

[45] Wu J. T., Leung K., Leung G. M. (2020). Nowcasting and forecasting the potential domestic and international spread of the 2019-nCoV outbreak originating in Wuhan, China: a modelling study. *The Lancet*.

[46] Gao C., Liu J. (2013). Modeling and restraining mobile virus propagation. *IEEE Transactions on Mobile Computing*.

[47] Grobusch M. P., Weld L., Goorhuis A. (2021). Travel-related infections presenting in Europe: a 20-year analysis of EuroTravNet surveillance data. *The Lancet Regional Health - Europe*.

[48] CDC (2022). U.S. Mpox case trends reported to CDC centers for disease control and prevention. https://www.cdc.gov/poxvirus/monkeypox/response/2022/mpx-trends.html.

[49] CDC (2022a). Outbreak cases and data, centers for disease control and prevention. https://www.cdc.gov/poxvirus/monkeypox/response/2022/index.html.

[50] Reuters (2022). Hong Kong to start monkeypox vaccination on October 5. https://www.reuters.com/world/asia-pacific/hong-kong-start-monkeypox-vaccination-october-5-2022-09-21/.

[51] Monkeypox 2022 Global Epidemiology; Report 2022-09-23 (2023). https://www.monkeypox.global.health/.

[52] De Baetselier I., Van Dijck C., Kenyon C. (2022). Retrospective detection of asymptomatic monkeypox virus infections among male sexual health clinic attendees in Belgium. *Nature Medicine*.

[53] Isaacs S. N. (2022). Asymptomatic infection? another reason to consider monkeypox a disease of public health concern. *Annals of Internal Medicine*.

[54] Ferré V. M., Bachelard A., Zaidi M. (2022). Detection of monkeypox virus in anorectal swabs from asymptomatic men who have sex with men in a sexually transmitted infection screening program in Paris, France. *Annals of Internal Medicine*.

[55] Gao C., Fan Y., Jiang S., Deng Y., Liu J., Li X. (2022). Dynamic robustness analysis of a two-layer rail transit network model. *IEEE Transactions on Intelligent Transportation Systems*.

